# Intramedullary Spinal Hemorrhage in Behcet’s Syndrome: A Case Report and Systematic Review

**DOI:** 10.7759/cureus.47134

**Published:** 2023-10-16

**Authors:** Ankita Das, Sima Vazquez, Eris Spirollari, Jose Dominguez, Merritt D Kinon, John K Houten

**Affiliations:** 1 Neurosurgery, New York Medical College, Valhalla, USA; 2 Neurosurgery, Westchester Medical Center, Valhalla, USA; 3 Neurosurgery, Icahn School of Medicine at Mount Sinai, New York City, USA

**Keywords:** spinal, hemorrhage, spine, vasculitis, behcet’s

## Abstract

Acute neurological manifestations in patients with Behcet’s syndrome are rare yet may lead to devastating outcomes. Distinguishing primary neurological deficits from spontaneous hemorrhagic insults is of particular importance for the prognosis of patients with Behcet’s syndrome. Here, we investigate the clinical characteristics, management, and outcomes of nontraumatic hemorrhagic injury in patients with Bechet’s syndrome. Following the case presentation, a systematic review of the literature identified cases of spontaneous hemorrhage among patients with Behcet’s syndrome. Variables of interest were collected from each article to characterize patient demographics, clinical manifestations, management, and reported outcomes. Additionally, a rare case of nontraumatic intramedullary spinal bleeding in a young male with Behcet’s syndrome is presented. Including our case, we analyzed 12 cases of spontaneous bleeding associated with Behcet’s syndrome in 12 articles. Patient age ranged from 16 to 71 (median = 36), with a male predominance (n = 11, 91.7%). Involvement of cardiothoracic structures (n = 3, 25%), pulmonary (n = 4, 33.3%), and gastrointestinal or genitourinary vasculature (n = 3, 25%) was most common, followed by extracranial (n = 2, 16.7%) and central nervous system vasculature (n = 1, 8.3%). Clinical presentation varied depending on which specific systems or anatomical structures were involved. Anticoagulation or antiplatelet therapy was mentioned in three cases (27.3%). Erythrocyte sedimentation rate (ESR) or C-reactive protein (CRP) were noted to be elevated in six cases (54.5%). Most cases were managed surgically (n = 8, 66.7%); four cases were managed conservatively (33.3%). In our case, the patient’s intramedullary bleed was allowed to dissolve without further manipulation. Of the reported outcomes, major recovery was achieved in 10 patients (83.3%), and two patients died from aneurysm or pseudoaneurysm rupture (16.7%). New-onset neurological findings in patients with Behcet’s syndrome should raise suspicion for possible spontaneous hemorrhage. Our case presents the first reported instance of an abrupt onset of neurological injury secondary to intramedullary spinal cord bleed in Behcet’s syndrome. A systematic review of the literature demonstrates no difference in mortality for patients managed conservatively compared to those who undergo surgical treatment.

## Introduction

Behcet’s syndrome (BS) is a systemic vasculitis affecting arterial and venous vessels of all sizes, resulting in a broad array of clinical manifestations such as aphthous and genital ulcers, ocular uveitis, skin lesions, arthritis, and musculoskeletal or gastrointestinal discomfort [1-3]. The variability in presentations is particularly prominent given its relapsing and remitting course, similar to other conditions with a presumed auto-immune basis [4,5]. Neurological involvement is rare, but when it occurs, it is often accompanied by severe disability and significant morbidity and mortality [6,7].

Neuro-Behcet’s Disease (NBD) has been coined for the condition in which neurological symptoms, such as headaches, migraines, meningoencephalitis, aneurysms, dissections, or dural sinus thrombosis, predominate [8]. According to one study, spinal cord lesions have been reported in up to 10% of NBD, while postmortem investigations revealed spinal lesions in 28% of cases [7]. A review of spinal cord involvement identified longitudinal transverse myelitis as the most common manifestation, with patients experiencing concomitant sensory deficits, bowel or bladder dysfunction, and back pain [9]. The variations in etiology and heterogeneity of the clinical features of patients with spinal cord involvement have sometimes led to late diagnosis or misdiagnosis, often subsequently leading to a poor prognosis [10]. Given these challenges, we performed a systematic review of the literature to further investigate the clinical characteristics, management, and outcomes of spontaneous hemorrhagic injury in patients with BS. In addition, we present an illustrative, rare case of a patient with BS with acute-onset back pain and lower extremity neurological deficits found with an intramedullary spinal cord hemorrhage.

## Case presentation

History

A 19-year-old who emigrated from Egypt at age nine had been diagnosed with BS five years before the current presentation. For the first three years from symptom onset, he experienced recurring cutaneous skin lesions identified as erythema nodosum but did not report many other complaints. After three years, he developed severe chest pain and was found to have multiple pulmonary emboli and a large right ventricular thrombus, for which he received a thoracotomy and a thrombectomy. He was subsequently placed on warfarin and had not experienced any further major complications until this present episode. Finally, he presented to the emergency department with abrupt-onset back pain, bilateral lower extremity weakness, and lower abdominal and bilateral lower extremity sensory loss, which was greater on the right side. Over the course of the past year, he has experienced frequent oral lesions, eruptions of erythematous papules, prolonged fevers, blurry vision with scleral injection, and a blood-tinged cough.

Physical examination and work-up

On neurological examination, he appeared to be a well-nourished, appropriately developed young male grimacing and complaining of mid-back pain. In terms of the Medical Research Council Muscle Power Assessment, which ranges from zero (no movement) to five (normal power), the right lower extremity demonstrated one out of five power (flicker of movement), while the left lower extremity demonstrated four out of five power (submaximal movement against resistance). Sensation was diminished bilaterally, but to a greater degree on the right. Deep tendon reflexes were normal. For the plantar reflex, the left toe was down-going, but the right was muted, demonstrating consistently greater deficits on the right side. He had lost his rectal tone.

**Figure 1:**
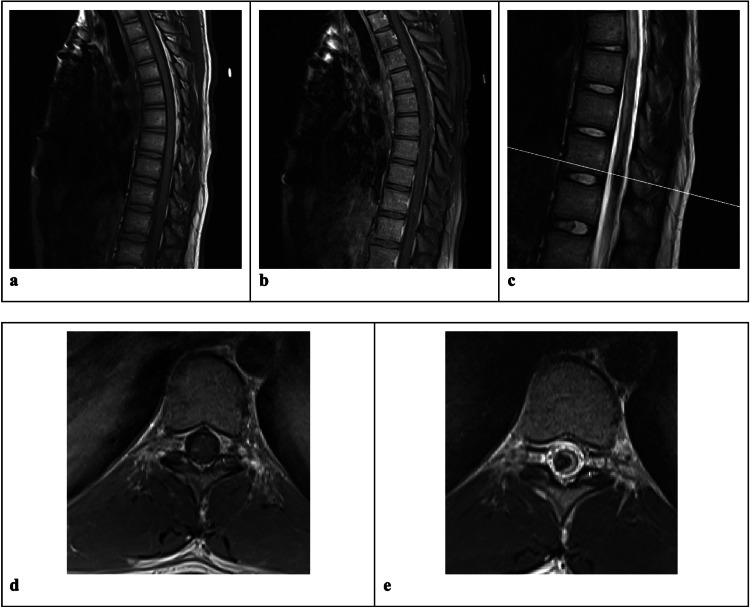
Imaging at presentation Sagittal T1 MRI (a) and sagittal T1+ MRI with contrast (b) demonstrate enhancement of an intramedullary spinal cord lesion. Sagittal T2 MRI (c) demonstrates the presence of hyperintensity cranial and caudal to the lesion, suggesting edema. Axial T1 MRI (d) and axial T2 MRI (e) reveal lateralization of the lesion to the right.

MRI of the thoracic spine revealed findings most consistent with intramedullary hemorrhage eccentric to the right hemi-cord with extensive surrounding cord edema (Figure 1). There were no abnormal vessels in the subarachnoid space to raise suspicion for a dural arteriovenous fistula or arteriovenous malformation. The decision was made not to operate due to the intramedullary nature of the bleed. To promote the best neurological outcome, the team chose to allow the bleed to dissolve without any further manipulation.

Laboratory evaluation was notable for elevated C-reactive protein (CRP), and biopsy of his skin lesions revealed neutrophilic dermatosis. A coagulation profile showed a prolonged prothrombin time (PTT) of 41.4 seconds, an activated partial thromboplastin time (aPTT) of 62.4 seconds, and an international normalized ratio (INR) of 3.6.

Follow-up

At six-month follow-up, the patient had persisting paraplegia with no appreciable improvement in lower extremity motor or sensory deficits. He remained incontinent of bowel and bladder. To balance the risk of his prior thrombotic complications, but given the recency of the spinal bleed, he was restarted on anticoagulation two weeks after the bleed at a lower INR goal of 1.5-2, an adjustment from his previous regimen maintenance goal of 2.5-3.

Systematic review

Search Strategy and Eligibility

**Figure 2:**
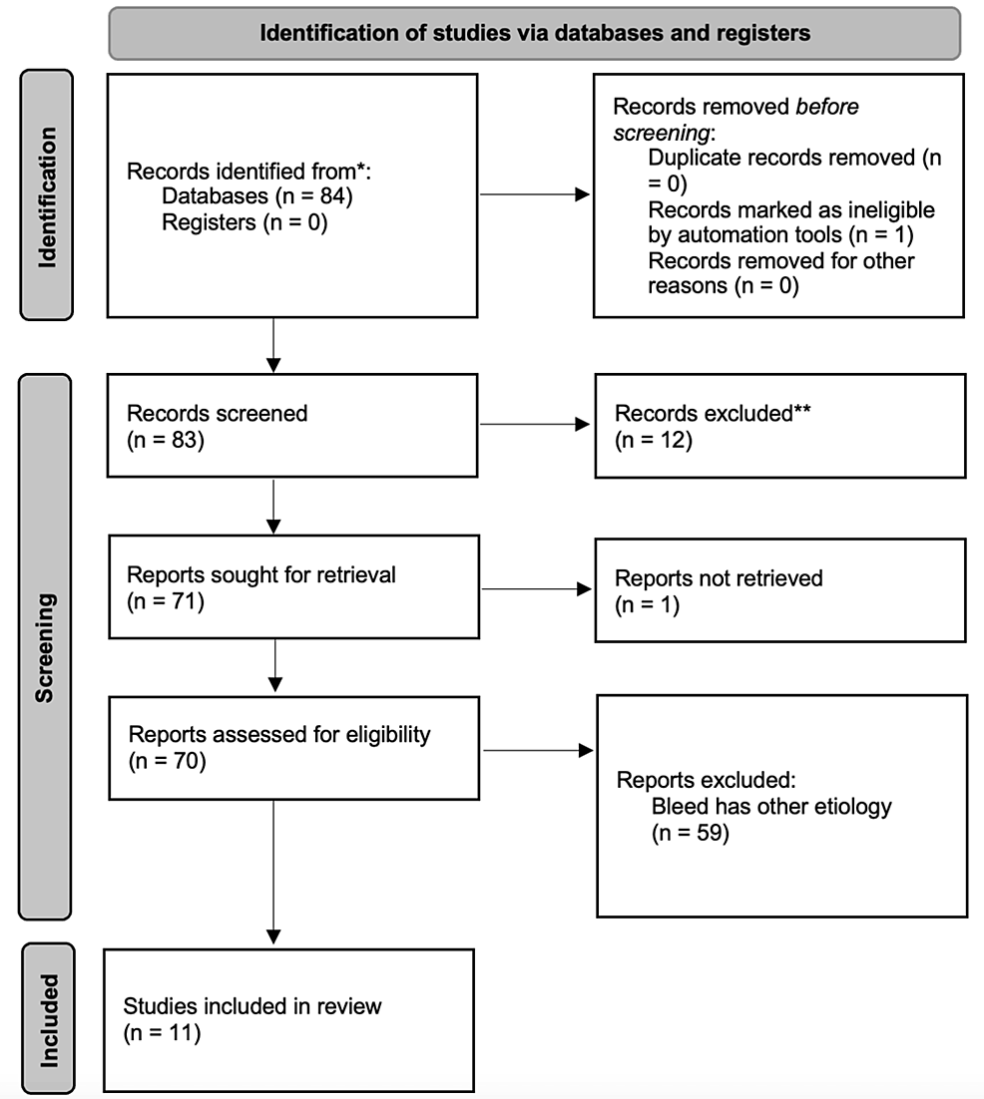
PRISMA diagram (PRISMA 2020).

A search query on PubMed was performed on July 8, 2021, using the following search terms: "spontaneous, hemorrhage, and behcet." The inclusion criteria were as follows: (1) A spontaneous or non-traumatic bleed was reported. The exclusion criteria were as follows: (1) Article not available in English; (2) Not a full article; (3) Bleed has other etiology. In accordance with the PRISMA 2020 guidelines, a diagram was generated for the results (Figure 2).

Data Collection and Variables

Pertinent data was extracted from each study that satisfied the inclusion and exclusion criteria. Individual studies were ordered based on the publication date. Patient age, sex, clinical manifestations, status of anticoagulation therapy use, acute phase reactant values, diagnostic tests utilized, treatments incorporated, and follow-up course were collected from each study. Selection of conservative or operative management and patient mortality were the primary outcomes measured.

Statistical Analysis

Patient demographics and clinical characteristics were compared between patient groups depending on whether conservative or operative management was performed and whether a patient died or lived, as described in each study. IBM Corp. Released 2021. IBM SPSS Statistics for Windows, Version 28.0. Armonk, NY: IBM Corp was utilized to complete the statistical analysis. The Shapiro-Wilk-Wilk for normal distribution was utilized to assess continuous variables. The student's t-test and the Mann-Whitney U test were also used to compare continuous variables that were normally and non-normally distributed. Measures of patient age were provided through descriptive statistics. Median patient age, defined as 36 years old, was used to stratify patients into a categorical variable. Pearson's χ2 test or Fisher's exact test was used to compare categorical variables, as appropriate.

Article Selection

Eleven articles met inclusion and exclusion criteria (Figure 1). All 11 were case reports describing spontaneous or nontraumatic bleeds in patients with Behcet's syndrome and are outlined in Table 1. Including our case, there were 12 patients total. Eleven were male, one was female, and the median age was 36. Six patients had elevated erythrocyte sedimentation rate (ESR) or CRP, and three patients were on anticoagulation (Tables 2, 3).

**Table 1:** Systematic review article summaries

	Author	Title (Year)	Age (Year)	Sex	Manifestations	Anti- coagulation	Acute Phase Reactants/ ESR Δ	Diagnostics	Treatment	Follow-Up Course
1	Lee et al. [9]	Spontaneous aortic pseudoaneurysm rupture into the sigmoid colon in Behcet’s disease patient (2015)	37	M	Recurrent oral & genital ulcers, hematochezia, tenderness of lower abdomen	N/A	CRP 0.94 mg/dL	Sigmoidoscopy: submucosal tumor-like projection with ulceration in sigmoid colon CT with enhancement: lower GI bleeding, enhanced pseudoaneurysm above the site of inferior mesenteric artery	Stent grafting through femoral artery	4 days: sigmoidoscopy demonstrated no active bleeding 6 months: Complete remission of pseudoaneurysm without graft infection
2	Roguin et al. [11]	A fatal case of Behcet’s disease associated with multiple cardiovascular lesions (1997)	26	M	Fever of unknown origin, chest pain, dyspnea, cough, hemoptysis, pulmonary opacity	N/A	ESR 87 mm/h	Lung biopsy: hemorrhagic infarct, occluded pulmonary arteries Echo: large pedunculated mass in the right atrium	LA mass was excised surgically Prednisone	8 weeks after initial admission, the patient died from bleeding of ruptured ascending aortic pseudoaneurysm
3	Chen et al. [12]	Right sinus of Valsalva aneurysm spontaneously dissecting into the interventricular septum in a rare case of Behcet’s disease (2019)	44	M	Cardiac murmur, recurrent oral-genital aphthous ulcers, rash	N/A	Minimal rise in CRP/ESR	ECG: poor R-wave progression across anterior leads TTE: perforation of the sinus of the Valsalva aneurysm (“perivalvular cystic-like mass"). CT/CTA: Confirmation	High dose steroids & immunosuppressants	N/A
4	Kim et al. [13]	Subepicardial hematoma compressing the right atrium: spontaneous rupture of the right coronary artery (2008)	28	M	Chest pain, shortness of breath	N/A	N/A	Echocardiography: right atrial pericardial mass CT: cystic mass in the atrioventricular groove CTA: occlusion of the R. coronary artery	Evacuation of hematoma with assistance of cardiopulmonary bypass	Discharged on postoperative day 10, postoperative echocardiogram shows no filling defect
5	Salamon et al. [14]	Massive hemoptysis complicating Behcet’s syndrome: the importance of early pulmonary angiography and operation	16	M	Recurrent hemoptysis for 2 wks	Long term heparin. Was discontinued and switched to warfarin.	ESR 100 mm/hr	Lobe excision: intrabronchial bleeding Pulmonary artery aneurysm	Lobe excision	Patient was well at 12 months f/u with no further episodes of hemoptysis
6	Barberis et al. [15]	Massive Haemoptysis in Behçet Syndrome: A Case Report (1987)	31	M	Recurrent oral and genital ulcerations, conjunctivitis, thrombophlebitis, necrotizing vasculitis of pulmonary vasculature	N/A	CRP positive, increased fibrinogen (420 mg/dL)	Biopsy: thrombophlebitis of erythema nodosum-like lesions Chest X-ray: Pulmonary infiltrates Autopsy: rupture of aneurysm of the pulmonary artery	Increased dosage of corticosteroids	Became asymptomatic and was discharged 1 week later, died 6 months later from massive hemoptysis
7	Wang et al. [16]	Behcet’s Syndrome Complicated by Multiple Aneurysms with Rupture, Hemorrhage, and Infection: A Case Report (1985)	52	M	Iritis, oral ulcers, gential ulcers, vision loss, glaucoma, profuse bleeding post-biopsy, popliteal fossa + groin + chest mass, upper limb numbness, wrist drop	N/A	N/A	A biopsy of the chest “mass: resulted in hemorrhage. Angiogram: axillary, popliteal, and femoral aneurysms Pus culture: + for staphylococcus, Klebsiella, and enterococcus	Subclavian artery opened, infected portion with thrombi removed Popliteal aneurysmectomy Resection of femoral artery aneurysm	Antibiotics course, discharged 30 days after admission, full recovery at 6 months, improvement of wrist drop
8	Orhan et al. [17]	Behcet’s disease and spontaneous haematocele: an unusual complication (1999)	37	M	Painful, right scrotal swelling	None	N/A	Ultrasound: echogenic mass within scrotum	Emergency exploration revealed haematocele from ruptured vein within right pampiniform plexus	Pt was discharged on day 2 post-op
9	Wu et al. [18]	Spontaneous Intra-Abdominal hemorrhage due to rupture of jejunal artery aneurysm in Behcet Disease (2015)	35	M	Paroxysmal abdominal pain, nausea, vomiting, hematochezia, recurrent aphthous oral ulceration, erythema nodosum on lower limbs	Provided good response	CRP/ ESR not within normal ranges (ESR = 40 mm/h), CRP = 60 mg/L)	CT: aneurysm of the superior mesenteric artery with thrombosis Contrast-enhanced CT: distal jejunal artery aneurysm, splenomegaly, renal infarction CBC: Drop in hematocrit	Resection of intestinal mass, formation of intestinal anastomosis	Follow up CT showed return of blood flow to visceral arteries, anti-inflammatory showed good response over course of 8 months
10	Bahar et al. [19]	Spontaneous dissection of the extracranial vertebral artery with spinal subarachnoid haemorrhage in a patient with Behcet’s disease (1993)	40	M	Acute headache, vomiting, confusion, stiff neck, right facial weakness, left hemiparesis, dysarthria, truncal ataxia	N/A	ESR 60 mm/h	MRI: Right pontine hyperintense lesion on T2 MRI CT Angiogram: Dissection at the V2 segment of the right vertebral artery	High-dose methylprednisolone	Steady improvement, discharged 1 month later with only slight truncal ataxia
11	Seo et al. [20]	Nontraumatic Subperiosteal Orbital hematoma in a patient with Behcet Disease (2020)	71	F	Periorbital pain, proptosis, diplopia	N/A	N/A	Contrast enhanced CT: mass in extra coronal superior left orbit found to be subperiosteal hematoma	Surgical exploration, hematoma and intra-cystic lesion evacuated	Eye range of motion improved 2 months post-operative follow-up: no residual hematoma 12 months: no residual symptoms
12	Our case	Intramedullary Spinal Hemorrhage in the Context of Behcet Syndrome: A Case Report and Systematic Review	19	M	Abrupt onset back pain and right lower extremity more than left extremity weakness and sensory loss	Warfarin, (prolonged PT 41.4 seconds, aPTT 62.4 seconds, INR of 3.6)	Elevated CRP	MRI thoracic spine: intramedullary hemorrhage eccentric to the right hemicord with extensive surrounding cord edema	The team chose to allow the bleed to dissolve without any further manipulation	

**Table 2:** Baseline characteristics stratified by management § OR: Odds ratio; CI: Confidence interval; ESR: Erythrocyte sedimentation rate; CRP: C-reactive protein

Variables	All (n = 12)	Operative Management (n= 8, 66.7%)	Conservative Management (n = 4, 33.3%)	Conservative Management		Operative Management	
				OR (CI 95%) §	P-value	OR (CI 95%)	P-value
Demographics		n (%)	n (%)				
Male	11 (91.7%)	7 (87.5%)	4 (100%)	0.875 (0.673-1.137)	1	1.143 (0.88-1.485)	1
Female	1 (8.3%)	1 (12.5%)	0 (0%)	0.875 (0.673-1.137)	1	1.143 (0.88-1.485)	1
Age Greater Than Median (36 y.o.)	6 (50%)	4 (50%)	2 (50%)	1 (0.091-11.028)	1	1 (0.091-11.028)	1
Elevated ESR or CRP §	6 (54.5%)	3 (42.9%)	3 (75%)	4 (0.265-60.325)	0.545	0.5 (0.031-7.994)	1
Anticoagulation	3 (27.3%)	2 (28.6%)	1 (25%)	0.833 (0.051-13.633)	1	0.667 (0.037-11.936)	1
Presentation							
Cardiothoracic Structures	3 (25%)	1 (12.5%)	2 (50%)	7 (0.397-123.347)	0.491	1 (0.063-15.988)	1
Pulmonary Vasculature	4 (33.3%)	3 (37.5%)	1 (25%)	0.556 (0.038-8.085)	1	1.8 (0.124-26.196)	1
Gastrointestinal or Genitourinary	3 (25%)	3 (37.5%)	0 (0%)	0.625 (0.365-1.069)	0.491	1.6 (0.935-2.737)	0.491
Extracranial	2 (16.7%)	1 (12.5%)	1 (25%)	2.333 (0.107-50.982)	1	0.429 (0.02-9.364)	1
Central Nervous System	1 (8.3%)	0 (0%)	1 (25%)	1.333 (0.757-2.348)	0.333	0.75 (0.426-1.321)	0.333

**Table 3:** Outcomes and morbidity § OR: Odds ratio; CI: Confidence interval; ESR: Erythrocyte sedimentation rate; CRP: C-reactive protein

Variables	All (n = 12)	Recovered (n = 10, 83.3%)	Died (n = 2, 16.7%)	Died	
				OR (CI 95%) §	P-value
Demographics		n (%)	n (%)		
Male	11 (91.7%)	9 (90%)	2 (100%)	0.9 (0.732-1.107)	1
Female	1 (8.3%)	1 (10%)	0 (0%)	0.9 (0.732-1.107)	1
Age Greater Than Median (36 y.o.)	6 (50%)	6 (60%)	0 (0%)	0.4 (0.187-0.855)	0.455
Conservative Management	4 (33.3%)	3 (30%)	1 (50%)	2.333 (0.107-50.982)	1
Operative Management	8 (66.7%)	7 (70%)	1 (50%)	0.429 (0.02-9.364)	1
Elevated ESR or CRP §	6 (54.5%)	5 (50%)	1 (100%)	0.5 (0.269-0.929)	1
Anticoagulation	3 (27.3%)	3 (30%)	0 (0%)	0.7 (0.467-1.05)	1
Presentation					
Cardiothoracic Structures	3 (25%)	2 (20%)	1 (50%)	4 (0.167-95.756)	1
Pulmonary Vasculature	4 (33.3%)	2 (20%)	2 (100%)	0.2 (0.058-0.691)	0.091
Gastrointestinal or Genitourinary	3 (25%)	3 (30%)	0 (0%)	0.7 (0.467-1.05)	1
Extracranial	2 (16.7%)	2 (20%)	0 (0%)	0.8 (0.587-1.091)	1
Central Nervous System	1 (8.3%)	1 (10%)	0 (0%)	0.9 (0.732-1.107)	1

Structures Involved

Three cases involved cardiothoracic structures, and four involved pulmonary structures [11-16]. Three cases described gastrointestinal or genitourinary vasculature [9,17,18]. Two involved the extracranial vasculature of the head [19,20.] Our case is the only one that describes central nervous system (CNS) involvement. There were no significant associations between the systems involved and management or outcomes (Tables 1, 2).

Anticoagulation/Antiplatelet Therapy

Anticoagulation or antiplatelet therapy was detailed in three cases, including ours. One case specified the absence of long-term anticoagulation therapy in the patient's long-term medication regimen [14]. However, this patient was on warfarin [14]. Wu et al. relayed only the positive effects of anticoagulation therapy but did not specify the details of the regimen [18]. The remainder of the reports did not mention any anticoagulation use. Our patient was on a warfarin regimen. There were no statistically significant associations between anticoagulation use and management of hemorrhage or outcomes (Tables 1, 2).

Diagnostics and Treatment

Computed tomography (CT) with enhancement was utilized most consistently to identify the nature of the lesion, while MRI, angiography, and ultrasound were also frequently employed. For suspected cardiac-related abnormalities, the standard modalities with well-established sensitivity and specificity, such as electrocardiography and transthoracic echocardiography, proved useful.

Four patients were treated with conservative management, including antibiotics for possible infections and corticosteroids (Table 2). Eight patients underwent operative management; resection or stenting of an aneurysmal area was performed, if possible, followed by a course of antibiotics to eliminate possible infection and corticosteroids to reduce overall inflammation (Table 2). There were no significant associations between demographics or variables of presentation and conservative management or operative management (Table 1). There were also no significant associations between conservative or operative management and outcomes (Table 2).

Outcomes

The outcome of each patient varied based on the location, size of the mass or aneurysm, and progression of the disease at the time of detection or intervention (Table 3). Two patients died from massive bleeding [11,15]. One case from 1987 describes a case of Behcet’s with recurrent oral and genital ulcerations, conjunctivitis, thrombophlebitis, and lung opacities on imaging [15]. This patient was treated with corticosteroids but died six weeks later from what an autopsy showed to be a ruptured aneurysm of the pulmonary artery [15]. Another 1997 patient presented with chest pain, dyspnea, cough, hemoptysis, and a fever of unknown origin [11]. A lung biopsy showed hemorrhagic infarction and occluded pulmonary arteries, and an echocardiogram showed a large mass in the right atrium. The mass was excised, and prednisone was administered. However, he died eight weeks after admission from a ruptured ascending aortic pseudoaneurysm [11]. The rest of the patients, whether treated surgically or medically, recovered near full functionality without many long-standing deficits. 

## Discussion

This is a rare case of spontaneous intramedullary spinal cord hemorrhage in a patient with BS. BS is a vasculitic disease with mucocutaneous, ocular, arthritic, vascular, and neurological manifestations [21]. Neurological manifestations reported in the literature include meningoencephalitis, meningitis, parenchymal involvement, spinal cord involvement, isolated intracranial hypertension, cerebral venous sinus thrombosis, and cranial nerve neuropathy [22]. In a study of 50 patients who had neurological complications of BS, imaging showed white-matter lesions on bilateral hemispheres that correlated with clinical findings in most cases presenting with brainstem syndromes [22]. However, literature reporting acute-onset neurological findings in the setting of BS is scarce. Similar symptomatology and imaging findings were previously reported in a 21-year-old patient with right hand numbness and multiple periventricular, cortical, and dorsal spinal cord hyperintensities, yet the etiology of the lesions had not been clearly established [23]. In fact, a prominent and common differential for such a presentation is multiple sclerosis, which may present similarly with intermittent lesions dispersed throughout the body and spinal cord involvement. Here, we present a rare case of spontaneous intramedullary bleeding in a patient with Behcet’s who presented with abrupt-onset back pain with lower extremity weakness and sensory changes.

The existing literature documenting cases of spontaneous hemorrhages in patients with BS reflects the immense variety in the presentation of BS. The diversity of clinical manifestations proves challenging for early diagnosis; therefore, timely management is of paramount importance to compensate for the severity of progression at the time of presentation. While most cases involved genital and oral aphthous ulcers, as is expected with Behcet’s, in some patients, localized symptoms were the most salient component, while in other patients, systemic symptoms were the most pronounced feature. The ultimate prognosis of a hemorrhage in BS appears to be dependent on multiple critical decision points guided by the astute application of diagnostic modalities. Non-invasive, accessible indicators such as serum inflammatory markers may serve as a primary barometer of active pathology. Localized imaging specific to the presenting clinical features allows the most detailed characterization of lesions; for example, cardiac complaints were often followed by electrocardiography and transthoracic echocardiography. Often, further investigation seemed to reveal an ambiguous mass, which prompted a biopsy. In most cases, angiography revealed the presence of multiple aneurysms with highly variable distribution throughout the body. The primary targets of treatment involved addressing the instability of the vascular abnormalities and managing the systemic inflammation. Most cases involved a combination of surgical and medical intervention-either excision of the unstable vasculature structure or conservative observation with the support of anti-inflammatory agents to balance the systemic inflammatory response. Anticoagulation therapy may be applied when necessary but does not appear to be a critical aspect of management as it was not explicitly specified in most cases.

Together, this review reflects the indiscriminate inflammatory nature of Behcet’s pathophysiology. Vasculo-Behcet’s can arise in a venous or arterial setting and across multiple organ systems. An analysis of over 100 patients with Behcet’s found that vasculitis may be a substitute initial presentation instead of the classic triad of oral ulcers, genital ulcers, and eye lesions; for those with vascular lesions, eye involvement and a positive pathology test seemed to be most highly associated versus non-vascular Behcet’s [24]. While limited in their sensitivity and specificity, changes in ESR, CRP, and other acute-phase reactants were consistently identified, suggesting that it may be valuable to place greater emphasis on such diagnostic markers that are quickly obtained in order to promptly address such high-acuity situations as hemorrhages. Similarly, studies have found that although isolated cases of spinal cord lesions are rare, cerebrospinal fluid with a neutrophilic predominance may provide useful evidence to support the diagnosis of NBD [25,26]. In a manner similar to the utilization of ESR/CRR, the measurement of neutrophils in thrombo-inflammation may warrant the prioritization of simple diagnostic tools such as complete blood count panels. Other serological parameters, such as hemoglobin levels, red cell distribution width, and ratios of platelets to lymphocytes, have also been found to be useful predictors of BS [27]. For a disease with an ambiguous presentation, judicious application of these lesser-known parameters in conjunction with patterns in more conventional but often overlooked measures may yield a more favorable prognosis.

The goal of this review was to highlight the variety within the management of hemorrhages associated with BS and to delineate the outcomes related to conservative management of precariously located hemorrhages, particularly those involving the CNS. In cases where surgical intervention may not be possible due to accessibility or the nature of the bleed, immunosuppressants can be an effective tool for decreasing the size and progression of thrombi and aneurysms [28]. In this case, the patient’s symptoms were derived from what was ultimately determined to be an intramedullary bleed. While spontaneous hemorrhage has been reported in the spinal cord in the setting of systemic anticoagulation, these are exceptionally rare events, and it is probable that the hemorrhage, in this case, was promoted by underlying vascular derangements associated with BS [29]. As diagnosis and treatment appear to occur on a case-by-case basis, this review is intended to consolidate the findings of the present reports on BS to facilitate timely decision-making regarding medical and surgical management.

## Conclusions

This is the first confirmed case of an intramedullary spinal bleed in a patient with Behcet’s disease, outlining the clinical decision-making arc from diagnosis to treatment to resolution. New-onset neurological findings in a patient in the context of other Behcet’s-associated symptoms should prompt investigation for spinal hemorrhage and timely management via either surgical or medical means, depending on the exact location and progression of the lesion and the systemic profile of the disease.

Autoimmune processes are often under-recognized as they relate to neurology, and the workup can be extensive in an attempt to exhaust other, more common etiologies. Yet the speed at which spinal pathologies may progress to irreversible deficits or vascular compromise necessitates greater awareness of other contributing conditions, especially in patients who have known histories of related diseases. This proves especially critical in patients demonstrating neurological deficits where quick determination of neurosurgical intervention is necessary. Our review attempts to encapsulate and centralize the variety of presentations of Behcet's disease to promote ease of diagnosis.

## References

[1] Saip S, Akman-Demir G, Siva A (2014). Neuro-Behçet syndrome. Handb Clin Neurol.

[2] Mat C, Yurdakul S, Sevim A, Özyazgan Y, Tüzün Y (2013). Behçet's syndrome: facts and controversies. Clin Dermatol.

[3] Caporn N, Higgs ER, Dieppe PA, Watt I (1983). Arthritis in Behcet's syndrome. Br J Radiol.

[4] Bettiol A, Prisco D, Emmi G (2020). Behçet: the syndrome. Rheumatology (Oxford).

[5] Fanouriakis A, Tziolos N, Bertsias G, Boumpas DT (2021). Update οn the diagnosis and management of systemic lupus erythematosus. Ann Rheum Dis.

[6] Noel N, Bernard R, Wechsler B (2014). Long-term outcome of neuro-Behçet's disease. Arthritis Rheumatol.

[7] Al-Araji A, Kidd DP (2009). Neuro-Behçet’s disease: epidemiology, clinical characteristics, and management. Lancet Neurol.

[8] Ambrose NL, Haskard DO (2013). Differential diagnosis and management of Behçet syndrome. Nat Rev Rheumatol.

[9] Lee HS, Kim do Y, Shin HY, Choi YC, Kim SM (2016). Spinal cord involvement in Behçet's disease. Mult Scler.

[10] Liu HM, Dong C, Zhang YZ (2017). Clinical and imaging features of spinal cord type of neuro Behçet disease: A case report and systematic review. Medicine (Baltimore).

[11] Roguin A, Edoute Y, Milo S, Shtiwi S, Markiewicz W, Reisner SA (1997). A fatal case of Behçet’s disease associated with multiple cardiovascular lesions. International Journal of Cardiology.

[12] Chen J, Liang HN, Wu L, Dong SH, Li JH (2019). Right sinus of Valsalva aneurysm spontaneously dissecting into the interventricular septum in a rare case of Behcet's disease. Eur Heart J Cardiovasc Imaging.

[13] Kim KH, Choi JB, Kim KS (2008). Subepicardial hematoma compressing the right atrium: spontaneous rupture of the right coronary artery. Ann Thorac Surg.

[14] Salamon F, Weinberger A, Nili M (1988). Massive hemoptysis complicating Behçet’s syndrome: the importance of early pulmonary angiography and operation. The Annals of Thoracic Surgery.

[15] Barberis M, Casadio C, Borghini U (1987). Massive haemoptysis in Behçet syndrome: case report. Respiration.

[16] Wang ZG, Ma SZ, Sheng HM, Bai YX, Yang N (1985). Behçet's syndrome complicated by multiple aneurysms with rupture, hemorrhage and infection--a case report. Chin Med J (Engl).

[17] Orhan I, Onur R, Ardicoglu A, Salatan Y (1999). Behçet's disease and spontaneous haematocele: an unusual complication. BJU Int.

[18] Wu XY, Wei JP, Zhao XY (2015). Spontaneous intra-abdominal hemorrhage due to rupture of jejunal artery aneurysm in Behcet disease: case report and literature review. Medicine (Baltimore).

[19] Bahar S, Coban O, Gürvit IH, Akman-Demir G, Gökyiğit A (1993). Spontaneous dissection of the extracranial vertebral artery with spinal subarachnoid haemorrhage in a patient with Behçet's disease. Neuroradiology.

[20] Seo JW, Kim EH, Han SE (2020). Nontraumatic subperiosteal orbital hematoma in a patient with Behcet disease. J Craniofac Surg.

[21] Borhani Haghighi A, Pourmand R, Nikseresht AR (2005). Neuro-Behçet disease. A review. Neurologist.

[22] Kidd DP (2017). Neurological complications of Behçet's syndrome. J Neurol.

[23] Sivri M, Koplay M, Erdoğan H, Keleşoğlu KS (2015). Neuro-Behçet disease presenting with spinal cord involvement as sudden onset hypesthesia at hand. Spine J.

[24] Koç Y, Güllü I, Akpek G (1992). Vascular involvement in Behçet's disease. J Rheumatol.

[25] Kalra S, Silman A, Akman-Demir G (2014). Diagnosis and management of Neuro-Behçet's disease: international consensus recommendations. J Neurol.

[26] Emmi G, Becatti M, Bettiol A, Hatemi G, Prisco D, Fiorillo C (2019). Behçet's syndrome as a model of thrombo-inflammation: the role of neutrophils. Front Immunol.

[27] Tezcan D, Körez MK, Gülcemal S, Hakbilen S, Akdağ T, Yılmaz S (2021). Evaluation of diagnostic performance of haematological parameters in Behçet's disease. Int J Clin Pract.

[28] Yadav T, Saxena S, Dutt N, Garg P, Khera P (2021). Vanishing aneurysms in Behcet's disease - A case series. Monaldi Arch Chest Dis.

[29] Shaban A, Moritani T, Al Kasab S, Sheharyar A, Limaye KS, Adams HP Jr (2018). Spinal cord hemorrhage. J Stroke Cerebrovasc Dis.

